# Chitosan- and Gelatin-Based Composite Granular Hydrogels for Cartilage Tissue Regeneration

**DOI:** 10.3390/ijms27062889

**Published:** 2026-03-23

**Authors:** Neda Khatami, Pedro Guerrero, Koro de la Caba, Ander Abarrategi, Sandra Camarero-Espinosa

**Affiliations:** 1 BioSmarTE Lab, POLYMAT, University of Basque Country UPV/EHU, Av. de Tolosa, 72, 20018 Donostia-San Sebastián, Spain; neda.khatami@polymat.eu; 2 Center for Cooperative Research in Biomaterials (CIC biomaGUNE), Basque Research and Technology Alliance (BRTA), 20014 Donostia-San Sebastián, Spain; ander.abarrategi@ehu.eus; 3 BIOMAT Research Group, University of the Basque Country (UPV/EHU), Escuela de Ingeniería de Gipuzkoa, Plaza de Europa 1, 20018 Donostia-San Sebastián, Spain; pedromanuel.guerrero@ehu.eus (P.G.); koro.delacaba@ehu.eus (K.d.l.C.); 4 BCMaterials, Basque Center for Materials, Applications and Nanostructures, UPV/EHU Science Park, 48940 Leioa, Spain; 5 Department of Cell Biology and Histology, Faculty of Medicine and Nursing, University of Basque Country (UPV/EHU), 48940 Leioa, Spain; 6 IKERBASQUE, Basque Foundation for Science, 48009 Bilbao, Spain

**Keywords:** granular hydrogel, cartilage, tissue regeneration

## Abstract

Cartilage regeneration remains an unmet clinical challenge. Despite the great advances in the production of hydrogels as support matrices for cartilage regeneration, the resulting mechanical properties remain low. Granular composite hydrogels appear as ideal candidates due to their injectability and modularity in design. Here, we report on the fabrication and characterization of heterogeneous composite granular hydrogels based on methacrylated chitosan (CHIMA) and gelatin (GelMA) microparticles supported by an interstitial methacrylated alginate (ALMA) matrix. Microparticles were prepared by an oil-emulsion method and their size and morphology optimized, resulting in CHIMA and GelMA microparticles of 10.8 µm (95% CI 9.2, 13.1) and 115.8 µm (95% CI 107.5, 137.6) in diameter, respectively. The microparticles were mixed with ALMA and crosslinked to form granular hydrogels that demonstrated reduced swelling and weight loss. The storage modulus increased from 33 to 66.4 kPa for CHIMA/ALMA hydrogels and from 11.5 to 19.5 kPa for GelMA/ALMA hydrogels when the particle concentration increased from 10 to 50%, and was higher than traditional ALMA hydrogels. Hydrogels of 50:50 CHIMA:GelMA permitted a 6.6-fold increase in cell number after 28 days of culture, and promoted the chondrogenic differentiation of embedded mouse mesenchymal stem cells with a glycosaminoglycan deposition of over 15 µg and the expression of chondrogenic markers.

## 1. Introduction

Articular cartilage lesions caused by trauma or degenerative diseases such as osteoarthritis have very limited intrinsic healing capacity and are the leading cause of disability in the elderly population [1,2]. Clinical treatments have focused on cartilage-stimulation approaches—including autologous chondrocyte implantation (ACI), matrix-assisted ACI (MACI), and microfracture techniques—to promote tissue repair [3]. However, these treatments often result in the formation of fibrocartilage that does not match the native joint environment, particularly the high-demanding load-bearing capacity of hyaline cartilage. Thus, these treatments fail in the long term (37% at 15-year postoperative), demonstrating early osteoarthritis in approximately 50% of the cases [4]. Therefore, developing effective strategies to promote functional hyaline cartilage regeneration remains a clinical priority.

Over the last few decades, new tissue regeneration strategies that make use of scaffold materials or hydrogels that serve as cell carriers have been thoroughly investigated [5,6,7]. Loaded with primary chondrocytes or bone marrow-derived stem cells, these materials are required to promote a chondrogenic phenotype in encapsulated cells while providing certain mechanical resistance. Some studies contemplate the intrinsically anisotropic and multilayered structure of native mature hyaline cartilage by fabricating gradient structures that can promote a layer-specific cell differentiation and matrix deposition, aiming at generating a mature tissue analog [8,9,10,11,12]. Others are centered on the fabrication of isotropic materials where cells can differentiate and deposit an initial cartilage template that would later mature in vivo, reaching a spatially defined tissue structure and cell phenotype [13,14,15]. Among the broad palette of synthetic and natural polymers used to that end, chitosan, gelatin, and alginate stand out as meritorious candidates [16,17]. However, the mechanical properties of fabricated hydrogels remain low, with reported compressive moduli of 1% (*w*/*v*) alginate and CHIMA hydrogels of 269 and 13 kPa, respectively, and an elastic modulus of about 35 kPa for 10% (*w*/*v*) GelMA hydrogels, which ultimately promotes the establishment of a hypertrophic chondrogenic phenotype [18,19].

Granular hydrogels have gained significant attention in tissue engineering over the last decade due to their unique and modular design, which offers flexibility and adaptability [20,21,22]. Granular hydrogels are composed of densely packed hydrogel microparticles in a jammed state and can be embedded within a support matrix to form composite granular hydrogels [23]. Unlike homogeneous bulk hydrogels, granular hydrogels contain a load-bearing network; paths of particles transmitting stress that govern stability and flow, which usually result in shear-thinning properties, essential for 3D bioprinting and injectability. The microparticles provide a tunable substrate for cell expansion and confer tunable porosity within the hydrogel for cell inclusion and nutrient transport. Additionally, these microparticles are building blocks that can be adjusted in size and packing density, as well as mixed to modulate the physicochemical properties, including the porosity of the resulting gel.

Recently, granular heterogeneous hydrogels fabricated by sizing bulk methacrylated hyaluronic acid (HAMA) and sulphated HAMA hydrogels into microparticles, which were later enzymatically crosslinked, were used to retain growth factors that promoted the infiltration and chondrogenic differentiation of bone marrow-derived stem cells [24]. Granular hydrogels demonstrated their capability to promote high cell infiltration and the deposition of a cartilage-like ECM that stiffened over the culture period. Moreover, comparison between hydrogels formed from 20 µm or 150 µm microparticles demonstrated that hydrogels from smaller particles promoted higher cell infiltration and deposition of collagen II and glycosaminoglycans, key markers of chondrogenesis, probably due to a higher void interconnectivity. However, these hydrogels displayed a maximum compressive modulus of 40 kPa, still far from that of native cartilage. Similarly, tyramine-modified hyaluronic acid bulk hydrogels have been sized and used to form granular hydrogels via enzymatic crosslinking [15]. Chondrocyte-laden granular hydrogels demonstrated high stiffening upon in vitro culture over 63 days, reaching values of 200 kPa. In vivo, granular hydrogels prepared from microparticles of varying sizes demonstrated that small-diameter microgels (20 µm) promoted continuous tissue maturation with low immunogenicity, as compared to hydrogels with higher microparticle diameter (100 and 500 µm). Yet, the fabrication of stable granular hydrogels requires fine-tuned inter-particle cross-linking strategies, the introduction of microparticle surface functional groups, and complex fabrication processes that result in hydrogels with reduced mobility. Thus, granular hydrogels would profit from more scalable fabrication processes and the implementation of interstitial or support hydrogels to increase stability and ease processability.

Granular hydrogel composites emerged a few years ago as an alternative to granular hydrogels to facilitate fabrication methods and to provide one extra layer of complexity and modularity on the physicochemical and mechanical properties of the resulting gel [25]. Yet, the reports on their applicability to cartilage tissue regeneration remain scarce. Here, we exploited the modularity of granular composite hydrogels to create support matrices for cartilage regeneration, hypothesizing that a combination of selected materials would enable the fabrication of hydrogels with both chondrogenic potential and mechanical strength. Thus, stiff methacrylated chitosan (CHIMA) and bioadhesive methacrylated gelatin (GelMA) microparticles were prepared and combined with methacrylated alginate (ALMA) as an interstitial matrix with chondrogenic potential to form three-dimensional granular hydrogels for cartilage regeneration. The resulting hydrogels demonstrated improved storage modulus, shear recovery, and chondrogenic potential.

## 2. Results

### 2.1. CHIMA Microparticle Development Using the Oil Emulsion Method

Methacrylated chitosan, or CHIMA, was chosen as a material to prepare microparticles for the granular hydrogels due to the higher mechanical properties and lower degradation rate as compared to traditional GelMA-based granular hydrogels [26,27]. The fabrication of chitosan microparticles has already been described in the literature by emulsification methods [28]. The oil emulsion methods provide a facile and fast process to produce particles at large scales, but are subject to multiple variables such as polymer concentration, oil-to-sample ratio, oil-to-surfactant ratio, stirring speed, crosslinking type, and concentration. In order to define the optimal microparticle preparation conditions, chitosan microparticles (CS), instead of CHIMA, were developed using the oil emulsion method followed by a crosslinking step with sodium citrate (NC) or tripolyphosphate (TPP). Chitosan concentrations of 1%, 2%, 5%, and 8% were tested to optimize the process. The primary objective was to establish a reliable method for preparing chitosan microparticles, which would subsequently inform the development of CHIMA microparticles under the optimized conditions. Various chitosan microparticle preparation parameters were systematically varied to determine their impact on microparticle formation, using sodium citrate (Table S1) or TPP (Table 1).

**Figure 1:**
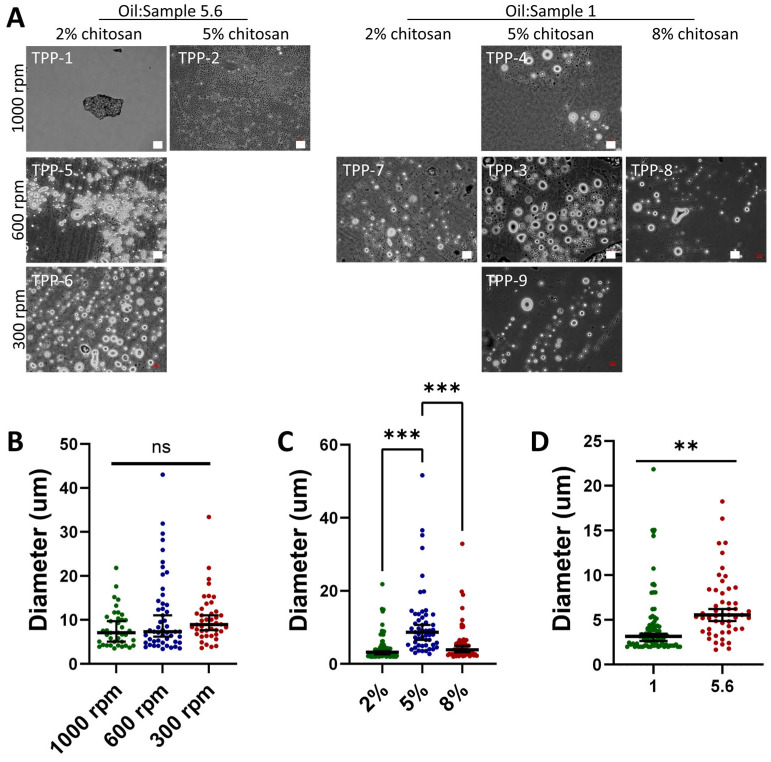
Chitosan microparticles prepared by emulsification under various process conditions. (**A**) Optical microscopy images of chitosan microparticles obtained from emulsification processes by varying the emulsion agitation speed (rpm), the oil-to-sample ratio, and the initial polymer concentration. The scale bar in all images is 10 µm. (**B**) Effect of agitation speed on microparticle diameter, as measured from samples TTP-4, TPP-3, and TTP-9. (**C**) Effect of initial chitosan concentration on particle diameter as determined from samples TPP-7, TPP-3, and TPP-8. (**D**) Effect of oil-to-sample ratio as determined from samples TPP-5 and TPP-7. Data in (**B**–**D**) represents the median and CI 95%. Statistical analysis was performed using one-way ANOVA for (**B**,**C**) and a student *t*-test in sample (**D**); (***) *p* < 0.001 and (**) *p* < 0.01, ns = no significant difference.

Microparticles were prepared and analyzed using optical microscopy to evaluate their size and morphology immediately after separation from the oil phase and subsequent redispersion in water to avoid potential aggregation upon freeze-drying (Figure 1A and Figure S1). As observed, the microparticles produced using sodium citrate as a crosslinker (NC1 to NC10) appeared to be agglomerated or forming flake-like structures and lacked a distinct shape, although they were freshly prepared. Attempts to improve microparticle formation by increasing polymer concentration (sample groups NC1–2 and NC3–5), varying the oil-to-sample ratio (NC7–9), oil-to-surfactant ratio (NC5–6), or crosslinker concentration (NC2–3) failed to result in a significant improvement. The resulting objects remained poorly structured and aggregated, and thus, further characterization was not conducted. Sodium citrate was used as the crosslinker in emulsification conditions NC1–10, which appeared to be ineffective in forming robust microparticles with well-defined shapes that would support the centrifugation and filtration steps without collapsing or losing their morphological integrity under our emulsification conditions. Nevertheless, this process has been reported to yield spherical microparticles [28,29] and, thus, we hypothesized that sodium citrate was ineffective in achieving sufficient ionic crosslinking regardless of the crosslinking time, perhaps due to a low diffusivity across emulsification media (oil) and through the chitosan itself under our specific set-up conditions. Additionally, the short emulsification and crosslinking times might have further contributed to the incomplete formation of microparticles despite being reported earlier by others [28,29].

To overcome these challenges, the crosslinker TPP was used in subsequent tests. Crosslinking with TPP provided a more controlled and efficient crosslinking process, as TPP is known for its ability to form strong ionic bonds with chitosan, resulting in enhanced microparticle stability and uniformity (samples TPP-1 to TPP-9). Optical microscopy images (Figure 1A) revealed distinctly formed microparticles with minimal agglomeration and well-defined shapes for samples TPP-3 to TPP-9, indicating successful crosslinking. The improvement in microparticle formation and structural integrity is likely due to several key changes made to the protocol. Firstly, replacing sodium citrate with TPP as the crosslinker provided more effective ionic crosslinking. TPP interacts with the amino groups of chitosan, forming strong ionic bonds, which enhance the mechanical stability and robustness of the microparticles. Secondly, extending the emulsification time to overnight allowed for more uniform droplet formation in the oil phase, resulting in microparticles with consistent sizes. The longer emulsification period ensures that the chitosan droplets are stable and well-dispersed before crosslinking begins. Finally, increasing the crosslinking time to 7 h provided sufficient time for TPP to interact thoroughly with chitosan, ensuring complete crosslinking. This extended duration likely contributed to the improved particle shape and the prevention of agglomeration.

The process was optimized by studying the impact of polymer concentration (TPP-1 and TPP-2; TPP-3, TPP-7, and TPP-8), oil-to-sample ratio (TPP-2 and TPP-4; TPP-5 and TPP-7), and stirring speed (TPP-1, TPP-5, and TPP-6; TPP-3, TPP-4, and TPP-9) (Figure 1). An increased agitation speed appeared to reduce the particle diameter, although not significantly, with agitations of 1000 rpm, 600 rpm and 300 rpm resulting on microparticles of 7.1 (5.1, 9.7) µm, 8.7 (6.6, 11.0) and 8.9 (7.7, 11.0) when samples were prepared from 5% chitosan solutions and an oil:sample ratio of 1 (Figure 1B). Variation in the initial concentration of chitosan used to prepare the microparticles failed to show any clear trend, with median microparticle diameters of 3.2 (2.6, 3.5), 8.7 (6.6, 11.0), and 3.9 (3.2, 4.9) for polymer concentrations of 2%, 5%, and 8%, respectively (Figure 1C). Variation in the oil-to-sample ratio had a clear impact on the capability to prepare well-defined microparticles. Sample conditions TPP-1 and TPP-2, with an oil-to-sample ratio of 5.6, a stirring speed of 1000 rpm, and a concentration of 2% and 5% chitosan, respectively, failed to yield microparticles. Comparison of samples TPP-2 and TPP-4, where only the oil-to-sample ratio was varied (agitation speed (1000 rpm) and polymer concentration (5%) constant) seem to indicate that for high agitation speeds, lower oil-to-sample ratios are needed to yield well-defined microparticles. Oil-to-sample ratios of 5.6, but lower agitation speeds of 600 rpm (TPP-5) and 300 rpm (TPP-6), enabled the formation of particles. A direct comparison of the effect of the oil-to-sample with conditions TPP-5 and TPP-7 using 5.6 and 1.0 oil-to-sample ratios, respectively, indicated that the higher the oil-to-sample ratio, the higher the microparticle diameter, yielding median microparticle diameters of 5.6 (4.9, 6.2) and 3.2 (2.6, 3.5), respectively (Figure 1D).

**Figure 2:**
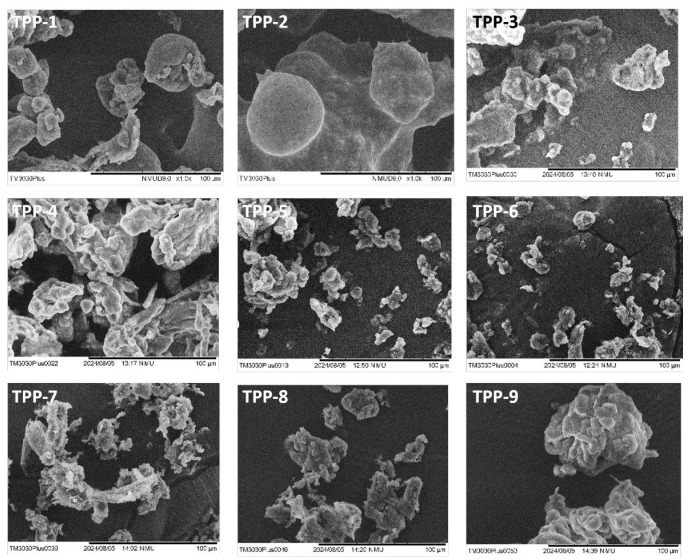
Chitosan microparticles prepared by emulsification under various process conditions. Optical microscopy images of chitosan microparticles obtained from emulsification processes by varying the emulsion agitation speed, the oil-to-sample ratio, and the initial polymer concentration. The scale bar in all images is 100 µm.

Scanning electron microscopy (SEM) was employed to further investigate the morphology and aggregation of the microparticles (Figure 2). While optical images revealed distinct, well-formed, and individualized microparticles, sample freeze-drying for SEM resulted in significant agglomeration. In SEM imaging, the samples are typically dried to avoid distortion under the vacuum. However, the drying process can cause microparticle shrinkage or aggregation, especially if they are highly hydrophilic or sensitive to moisture loss. It is also possible that the drying process, particularly if it involved rapid drying, contributed to aggregation, causing the particles to collapse or bind together. To mitigate this issue, the complete drying of the microparticles was avoided in the subsequent steps. Instead, the microparticles were used in their hydrated form for further analysis to prevent aggregation and preserve their intended morphology.

### 2.2. Development of Chitosan/ALMA Granular Hydrogel

**Figure 3:**
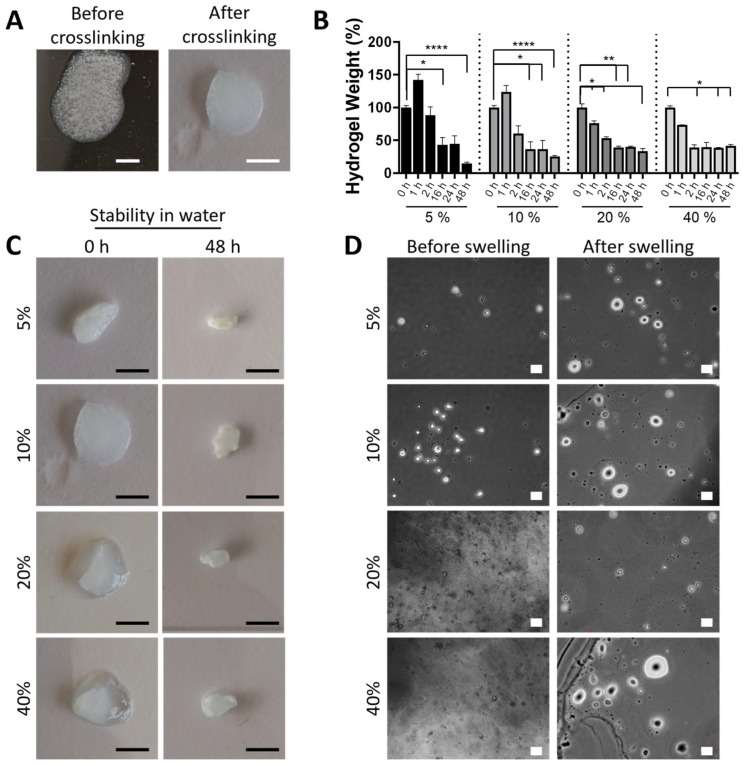
Stability of CS/ALMA granular hydrogels of varying microparticle concentration. (**A**) Photographs of CS/ALMA granular hydrogels with 10% (*w*/*v*) microparticle concentration before and after crosslinking, showing compactation and whitening of the resulting gel. Scale bar is 5 mm. (**B**) Weight loss of granular hydrogels of 5%, 10%, 20%, and 40% (*w*/*v*) microparticle concentration upon culture in liquid media over time. Data is presented as mean ± SD, *n* = 3. Statistical significance was calculated from two-way ANOVA with post hoc Tukey’s multiple comparison tests; (****) *p* < 0.0001, (**) *p* < 0.01, and (*) *p* < 0.1. (**C**) Photographs of CS/ALMA granular hydrogels with different microparticle concentrations before and after incubation in liquid media showing densification and mass loss. The scale bar is 5 mm in all pictures. (**D**) Optical microscopy images of CS/ALMA granular hydrogels after 48 h incubation in water, showing particle swelling. The scale bar is 20 µm in all images.

After optimization of the particle preparation process, the conditions from TPP-3 were selected for the development of chitosan-based and granular hydrogels, as these yield microparticles of the highest diameter, needed to fabricate cell-laden granular hydrogels. In this process, chitosan (CS) microparticles at concentrations of 5%, 10%, 20%, and 40% (*w*/*v*) were mixed with a 4% ALMA solution (*w*/*v*) to give rise to CS/ALMA hydrogels. Samples were prepared using a dual-crosslinking method based on ionic crosslinking, with CaCl_2_, and covalent photo-crosslinking with UV light through the methacrylate groups of ALMA. The initial microparticle dispersions were clear and with a liquid consistency, but retained the shape immediately after crosslinking, changing to a whitish gel structure (Figure 3A).

The stability of CS/ALMA granular hydrogels in water was evaluated over a period of 48 h (Figure 3B,C). Granular hydrogels with the lowest microparticle concentrations of 5% and 10% suffered swelling in the first hour of incubation, reaching a weight increase of 142 ± 9% and 124 ± 10%, respectively. This can be ascribed to the higher concentration of ALMA in the hydrogels, driving the overall swelling of the hydrogel. Overall, granular hydrogels suffered a weight loss of 41–58%, depending on the sample composition, over the first 16 h, which remained stable upon further incubation in water only in hydrogels with the highest concentration of microparticles. Samples with the lowest concentration of microparticles suffered the highest weight loss, with a remaining weight of approximately 14.5% and 25.4% for samples containing 5% and 10% microparticles, respectively. Samples with the highest microparticle concentration showed a remaining weight of approximately 33.5% and 41.5% for samples bearing 20% and 40% microparticle concentration. This weight loss can be ascribed to the densification effect observed visually (Figure 3C), by which the chitosan microparticles provided a structural reinforcement and improved network density. Chitosan is a polycation that can interact strongly with alginate, a polyanion, forming a polyelectrolyte complex that would increase the stability of the hydrogel [30]. In this system, ALMA undergoes dual crosslinking through ionic bonds facilitated by CaCl_2_ and covalent bonding induced by UV light. The CaCl_2_ promotes ionic crosslinking by interacting with the carboxylate groups of the alginate, creating a network of ionic bridges that enhance the hydrogel’s mechanical strength and stability in an aqueous environment. Simultaneously, UV light initiates the polymerization of methacrylate groups in the ALMA, forming covalent crosslinks that further stabilize the hydrogel structure. Although chitosan itself is not methacrylated and does not participate directly in the covalent crosslinking, its presence plays a significant role in reinforcing the hydrogel via complexation with alginate. The chitosan microparticles serve as a physical scaffold within the hydrogel matrix, increasing the packing density and reducing the mobility of the polymer chains. This physical reinforcement complements the crosslinking mechanisms of ALMA, resulting in a more robust hydrogel network that resists degradation more effectively. In fact, optical observation of the granular hydrogels before and after immersion in water for 48 h revealed an initial segregation of the microparticles within the structure (observed as a white and dense area) that became homogeneous over time as the excess, non-crosslinked material was washed away (Figure 3C). Upon incubation in water, chitosan microparticles also appeared to be swollen, with an increased diameter that could be observed by optical microscopy (Figure 3D).

### 2.3. Development of CHIMA/ALMA and GelMA/ALMA Granular Hydrogels

**Figure 4:**
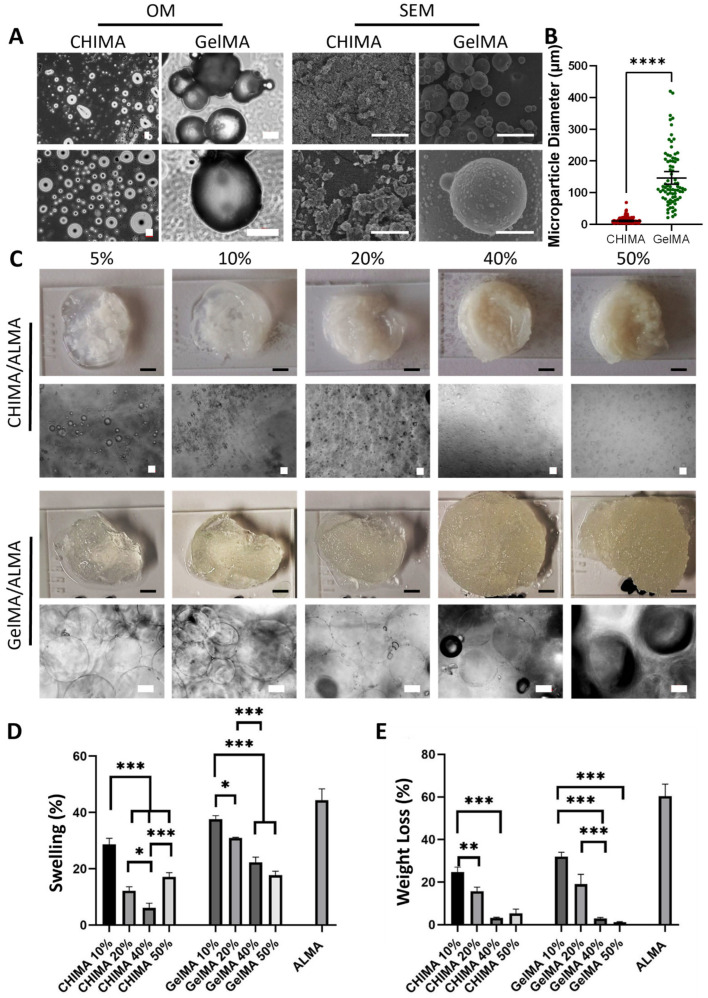
Stability of CHIMA/ALMA and GelMA/ALMA granular hydrogels of varying microparticle concentration. (**A**) Optical (OM) and scanning electron microscopy (SEM) images of CHIMA and GelMA microparticles. Scale bars are 20 µm and 100 µm for CHIMA and GelMA OM pictures, respectively, and 500 µm and 50 µm for the top and bottom rows of SEM images, respectively. (**B**) CHIMA and GelMA microparticle diameter as measured from OM images. Error bars represent median ± 95% CI. Statistical significance was calculated from a student *t*-test with (****) *p* < 0.0001, (***) *p* < 0.001, (**) *p* < 0.01, and (*) *p* < 0.1. (**C**) Pictures (top) and OM (bottom) images of CHIMA/ALMA and GelMA/ALMA granular hydrogels showing overall appearance and the microparticles within the gels. Scale bars are 10 mm for OM images, and are 20 µm and 100 µm for CHIMA and GelMA OM pictures, respectively. (**D**) Swelling of CHIMA/ALMA and GelMA/ALMA granular hydrogels, calculated as the weight increase after 72 h of incubation in water, and (**E**) the associated weight loss. Statistical significance was calculated from two-way ANOVA with post hoc Tukey’s multiple comparison tests; (****) *p* < 0.0001, (***) *p* < 0.001, (**) *p* < 0.01, and (*) *p* < 0.1.

To increase the stability of the developed granular hydrogels, chitosan was functionalized with methacrylate groups (CHIMA) that could polymerize with the bulk ALMA hydrogel (Figure S2). The inclusion of methacrylate moieties in the polymer is not expected to affect the size of the microparticles of the oil-emulsion process that remained unchanged for CHIMA microparticle production. Thus, CHIMA microparticles were obtained under the optimized conditions TPP-3 used for CS/ALMA hydrogels (Figure 4A), yielding slightly larger particles with a median diameter of 10.8 µm (95% CI 9.2, 13.1) as measured by optical and scanning electron microscopies (Figure 4B).

The development of granular hydrogels requires microparticles that are larger than 10 µm in diameter [31]. However, the formation of jammed structures with sufficient space for multicellular infiltration, growth, and matrix deposition requires particle diameters of at least 50 µm [31,32]. Therefore, we decided to combine CHIMA particles with particles of a larger diameter and higher bioadhesivity, using a protocol developed earlier by us for methacrylated gelatin (GelMA) microparticles [10]. The protocol makes use of a starting 30% (*w*/*v*) GelMA solution, and the obtained GelMA microparticles had a median diameter of 115.8 µm (95% CI 107.5, 137.6). A higher polymer concentration increases the viscosity of the aqueous phase, making it more resistant to shear forces during emulsification. As a result, it becomes more difficult to break the viscous polymer solution into smaller droplets within the oil phase, leading to the formation of larger microparticles. CHIMA microparticles were prepared using the oil emulsion method and then crosslinked with TPP after emulsification. The TPP crosslinking process stabilizes the droplets by chemically bonding the polymer chains, preventing further droplet coalescence or growth. This results in smaller, more uniform microparticles since the crosslinking locks the particle size at the point of emulsion formation. The ionic crosslinking with TPP ensures that the droplets are stabilized early in the process. In contrast, GelMA microparticles were created using a higher polymer concentration and then subjected to physical gelation in an ice bath. The higher concentration increases the viscosity of the emulsion, making it more difficult to break the polymer solution into smaller droplets. Additionally, since the gelation process is slower and dependent on temperature, the droplets tend to merge or grow before they are fully stabilized, leading to larger microparticles. The absence of chemical crosslinking during emulsification, combined with the higher concentration, allows for larger droplet formation that only begins to stabilize once the temperature drops in the ice bath.

Morphological characterization by SEM revealed GelMA microparticles with a distinct spherical shape and little aggregation (Figure 4A). In contrast, CHIMA microparticles appear to be agglomerated. Given that CHIMA microparticles were smaller than GelMA, they have a larger surface area relative to their volume, which makes them more susceptible to inter-particle interactions. As a result, these smaller particles are more likely to stick together or agglomerate during the drying process.

Next, both CHIMA/ALMA and GelMA/ALMA granular hydrogels were prepared using CHIMA and GelMA microparticles mixed and crosslinked with ALMA gel (Figure 4C). The goal was to enhance the crosslinking between the methacrylate groups of ALMA and the CHIMA or GelMA microparticles. In the case of GelMA/ALMA hydrogels, the microparticle formation involved physical crosslinking of the GelMA via chain entanglement, while chemical crosslinking was achieved through the methacrylate groups of GelMA and ALMA that strengthen the matrix, and using CaCl_2_ to crosslink the ALMA. The use of CaCl_2_, specifically for ALMA, promotes ionic crosslinking, contributing to the stability and integrity of the hydrogel network. For CHIMA/ALMA hydrogels, the process was similar to the GelMA-based system. However, in this case, the crosslinking primarily relied on ionic interactions between the chitosan in the CHIMA microparticles and TPP. Chitosan is a cationic polymer due to the presence of amino groups on its structure, which can form strong ionic bonds with negatively charged TPP molecules. Additionally, the methacrylate groups in both CHIMA and ALMA, as in the GelMA/ALMA system, undergo free radical polymerization upon UV exposure, creating a more robust, covalently bonded structure.

Different microparticle concentrations of 5%, 10%, 20%, 40%, and 50% were used to develop granular hydrogels (Figure 4C). Hydrogels loaded with low, 5–10% microparticle concentration presented phase segregation, with ALMA-rich and microparticle-rich phases that could be distinguished visually. Particularly, ALMA hydrogels loaded with 5% microparticle concentration lack structural stability and were discarded from further analysis. An increasing concentration of microparticles resulted in more whitish and opaque hydrogels for both CHIMA/ALMA and GelMA/ALMA. The larger size of GelMA microparticles was still evident after embedding in the ALMA hydrogel, especially when compared to CHIMA microparticles. It should be noted that as the microparticle concentration increased, the opacity and density of the granular hydrogels also increased, which caused light scattering during image acquisition and the difficulty of obtaining sharp, well-defined images of the embedded microparticles.

The results show that increasing the concentration of CHIMA microparticles generally leads to a decrease in the water absorption capacity of the hydrogels. This is likely because a higher microparticle content increases the density of the hydrogel structure, reducing the free space available for water uptake. However, an interesting observation is that hydrogels containing 50% microparticles exhibit higher water absorption compared to those containing 40% and 20% microparticles. This behavior could be explained by the possibility that, at very high microparticle concentrations, the particles themselves may act as additional hydrophilic domains, promoting water retention. Alternatively, the organization or packing of microparticles at high concentrations might create microvoids or interconnected pores within the hydrogel, which could facilitate greater water uptake despite the overall denser structure. CHIMA microparticles are less hydrophilic than pure chitosan or pure alginate but still have some moderate hydrophilicity due to remaining hydroxyl groups and incomplete methacrylation. However, this increased water uptake at 50% microparticle concentration is likely not due to the intrinsic hydrophilicity of CHIMA, which is moderate compared to highly hydrophilic polymers such as alginate. Rather, at very high microparticle concentrations, the network structure may become less densely packed, leading to the formation of pores and interstitial spaces within the hydrogel. These structural voids can physically trap more water, resulting in an overall higher swelling capacity despite the relatively lower hydrophilicity of the CHIMA microparticles themselves.

The structural stability of granular hydrogels upon culture in liquid media was evaluated for both CHIMA/ALMA and GelMA/ALMA systems (Figure 4D,E). Increasing the microparticle concentration within the hydrogels led to a reduction in water absorption. In fact, a correlation between water absorption density and a tighter network structure was created at higher microparticle loadings. As more microparticles are packed into the hydrogel matrix and crosslinked with the ALMA support polymer, fewer void spaces and interstitial gaps are available to accommodate water, thereby limiting the overall swelling capacity of the hydrogel. Interestingly, when the microparticle concentration was increased to 50%, the hydrogels exhibited a higher water absorption compared to the 40% and 20% microparticle formulations. This behavior could be due to the reduced packing efficiency at very high microparticle concentrations, which may create larger pores and a more heterogeneous network structure. These structural irregularities facilitate greater water penetration into the hydrogel matrix. Since the size of GelMA microparticles was approximately 10-fold that of CHIMA microparticles, a lower surface area and amount of methacrylic groups were available to bind ALMA; thus, the water absorption of GelMA/ALMA hydrogels was higher than that of CHIMA/ALMA hydrogels.

The weight loss of CHIMA/ALMA and GelMA/ALMA granular hydrogels after 72 h of incubation in water followed the same trend as for the water absorption (Figure 4E). Specifically, an increase in microparticle concentration led to enhanced hydrogel stability and reduced weight loss. Simultaneously, the lower water uptake and lower weight loss in hydrogels with higher microparticle concentrations also confirm a lower water content in these samples.

### 2.4. Viscoelastic Properties of CHIMA/ALMA and GelMA/ALMA Granular Hydrogels

**Figure 5:**
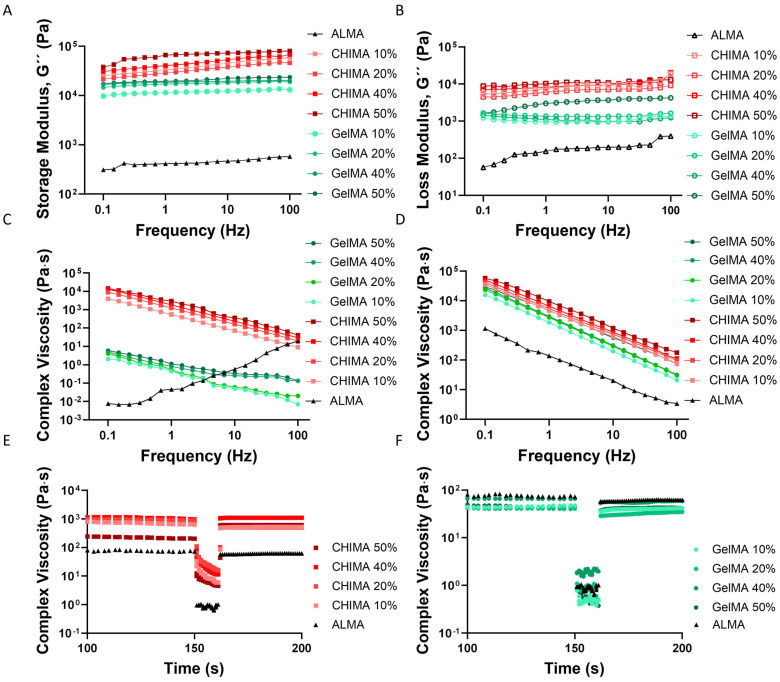
Viscoelastic properties of CHIMA/ALMA and GelMA/ALMA granular hydrogels with 0% (ALMA), 10%, 20%, 40%, or 50% microparticle concentration. Storage (**A**) and loss (**B**) moduli of CHIMA/ALMA and GelMA/ALMA granular hydrogels over a frequency sweep. Complex viscosity of CHIMA/ALMA and GelMA/ALMA granular hydrogels before (**C**) and after (**D**) crosslinking over a frequency sweep. Shear recovery of CHIMA/ALMA (**E**) and GelMA/ALMA (**F**) granular hydrogels.

Hydrogels for cartilage regeneration have traditionally been of low mechanical properties. Thus, the typical storage modulus of alginate-based hydrogels is in the order of a few hundred Pa, depending on the molecular weight [33]. These values are significantly lower than the reported storage modulus of human articular cartilage, which ranges from 32 to 43 MPa [34]. The inclusion of microparticles to form granular hydrogels has been shown to increase the storage modulus of the supporting matrix. The viscoelastic properties of CHIMA/ALMA and CHIMA/GelMA granular hydrogels were analyzed using rheological characterization. First, the loss and storage moduli of these hydrogels were analyzed to assess their overall mechanical performance and the effect of microparticle concentration on these properties (Figure 5A,B). The storage modulus (G′) reflects the elastic (solid-like) behavior of the hydrogel, representing its ability to store energy when deformed. In contrast, the loss modulus (G″) indicates the viscous (liquid-like) behavior, corresponding to the amount of energy dissipated during deformation. All granular hydrogels showed a significant increase in both storage and loss moduli as compared to ALMA hydrogels without any microparticles (Table 2). Thus, GelMA and CHIMA microparticles act as reinforcing fillers within the ALMA hydrogel, improving their capability to resist deformation and increasing both the elastic and viscous components of the material response under stress. Increasing microparticle concentration in both CHIMA/ALMA and GelMA/ALMA hydrogels led to increased G′ and G″. A higher concentration of microparticles results in a denser and more interconnected network within the hydrogel matrix. The microparticles not only occupy space but also provide additional physical crosslinking points or act as mechanical anchors, which restrict the mobility of the polymer chains. As a result, the hydrogel becomes stiffer (higher G′) and more resistant to deformation, while also exhibiting a slight increase in energy dissipation capacity (higher G″) under applied stress. This reinforcement effect is particularly important for applications where mechanical strength, durability, and resilience are critical, such as in the regeneration of articular cartilage.

CHIMA/ALMA granular hydrogels exhibited significantly higher storage and loss moduli compared to GelMA/ALMA hydrogels (Table 2 and Figure 5A,B). Granular hydrogels with 50% microparticle loading showed storage and loss moduli of 66.4 and 10.1 kPa and 19.5 and 2.9 kPa for CHIMA/ALMA and GelMA/ALMA hydrogels, respectively. This represents an approximate three-fold increase in both the elastic and viscous responses in CHIMA/ALMA compared to GelMA/ALMA hydrogels. The higher moduli observed in CHIMA/ALMA hydrogels likely result from the intrinsic properties of chitosan-based microparticles, which are stiffer and form stronger physical and ionic crosslinks within the network. In contrast, GelMA, derived from gelatin, is softer and more flexible, leading to lower mechanical resistance. While the storage modulus of human articular cartilage is in the range of 32 to 43 MPa [34], this is measured when a rich extracellular matrix is deposited by cells and further matures over time, acquiring the specific protein spatial organization observed in the tissue (e.g., collagen archades) and the stress-relaxation characteristic of water entrapping glycosaminoglycans. Thus, while the storage modulus reported here for granular hydrogels is not comparable to that of the native tissue, it might hinder the formation of the clasical hyperthophic tissue deposited in softer hydrogels.

The complex viscosity, a parameter derived from oscillatory rheology, reflects the resistance of a material to deformation under dynamic shear and encompasses both viscous and elastic contributions. It is particularly useful for evaluating the mechanical integrity of viscoelastic materials like hydrogels. The complex viscosity of the granular hydrogels before crosslinking showed a shear-thinning response in all compositions with an associated increase in viscosity with microparticle loading (Figure 5C). This behavior suggests a denser and more interconnected microstructure within the hydrogel, which resists flow more effectively due to the presence of a greater number of physical and chemical interactions between microparticles and the surrounding matrix. Shear-thinning is a desirable property for injectable or printable biomaterials, as it allows the material to flow under stress but recover its structure at rest. This behavior arises from the alignment and disentanglement of polymer chains and microparticles under shear, which temporarily reduces internal resistance to flow. Interestingly, ALMA hydrogels without microparticles show an opposite trend prior to crosslinking, with viscosity increasing under higher shear. This behavior may stem from the relatively low molecular weight and concentration of ALMA, leading to weak intermolecular interactions that shortly reorganize under shear stress. However, upon addition of either CHIMA or GelMA microparticles, the hydrogel matrix gains a more entangled and heterogeneous structure, restoring a typical shear-thinning response.

Photo-crosslinking strengthens the hydrogel by forming covalent or ionic bonds that reduce molecular mobility, thus greatly increasing resistance to deformation. Crosslinking of granular hydrogels resulted in an increased complex viscosity of approximately three orders of magnitude for GelMA and of five-fold for CHIMA, an approximate 33-fold and 69-fold increase with respect to ALMA alone on 50% microparticle loaded hydrogels, respectively (Figure 5D). This significant difference may be attributed to the inherent stiffness and positive charge density of chitosan-based particles, which interact more strongly with ALMA (negatively charged), leading to more effective stress distribution and reinforcement within the hydrogel network.

The physiological loading frequency of hyaline cartilage has been established to be in the range of 10^0^–10^3^ Hz, with the lowest frequencies of 10^0^–10^1^ Hz representing walking, up to 10^2^ Hz running or jumping and landing, and the highest, of up to 10^3^ Hz, representing a traumatic injury [35]. Our experimental setup represented physiological conditions (up to 10^2^ Hz). Recent studies of the rheological properties of bovine articular cartilage revealed clear differences between immature, mature, and pathological cartilage with storage moduli of approximately 2 MPa, 5 MPa, and 0.3 MPa, at 1 Hz, respectively, when a 5 N load was applied [36]. When a constant frequency of 1 Hz was applied, bovine cartilage displayed a storage modulus of 1 MPa at 0.01% strain. Our samples displayed a maximum storage modulus of 66 kPa for CHIMA/ALMA hydrogels at 50% microparticle loading and of 19 kPa for GelMA/ALMA samples at 50% loading. Thus, despite having an over 150-fold increase in the storage moduli of CHIMA/ALMA at 50% loading as compared to ALMA alone, the overall rheological properties of the developed granular hydrogels are still 1 order of magnitude below those of bovine articular cartilage under similar test conditions.

Shear recovery tests are used to assess the self-healing or structural resilience of hydrogels under dynamic mechanical stress, which is particularly relevant for applications involving injection, 3D printing, or load-bearing environments such as those of articular cartilage. The test involves subjecting the hydrogel to a high shear strain to disrupt its internal network, followed by monitoring the recovery of complex viscosity over time once the stress is removed. Cartilage is subjected to daily strains of up to 30%, and thus, the shear recovery was calculated as the percentage recovery of the complex viscosity from the initial, after a shear strain of 30% was applied to the sample [37]. The shape recovery capability of granular hydrogels increased with microparticle concentration for both CHIMA and GelMA hydrogels (Table 2 and Figure 5E,F). This improvement is likely due to the formation of a more physically entangled and densely packed network, where microparticles act as reversible physical crosslinkers or anchoring sites. These structures can temporarily deform under stress but reorganize quickly upon release, thus promoting recovery. Interestingly, hydrogels based on GelMA consistently exhibited slightly higher recovery percentages than their CHIMA counterparts at equivalent microparticle concentrations. For example, GelMA/ALMA hydrogels achieved a maximum recovery of 95.1%, compared to 91.6% and 90.2% for CHIMA/ALMA hydrogels with 40% and 50% microparticles, respectively. This difference may be attributed to the more flexible and hydrophilic nature of gelatin-derived particles, which facilitates faster chain reorganization and interaction with the ALMA matrix after shear. In contrast, CHIMA microparticles, derived from chitosan, are stiffer and more electrostatically interactive due to their cationic nature, potentially leading to a slightly slower or less efficient structural reformation. Moreover, the observation that CHIMA/ALMA with 50% microparticles had slightly lower recovery (90.2%) than the 40% group (91.6%) can be explained by the fact that at very high microparticle concentrations, the hydrogel may become excessively rigid or over-packed. This can reduce the mobility of polymer chains and limit the dynamic reformation of interactions required for full recovery, effectively compromising the reversibility of the internal network. Interestingly, even the ALMA hydrogel (0% microparticles) demonstrated a moderately high recovery capability, likely due to reversible physical interactions (e.g., hydrogen bonding or chain entanglements) within the polymer matrix.

Overall, the rheological analyses underscore the potential of granular hydrogels in applications such as bioprinting and cartilage tissue engineering, where mechanical performance is critical. The ability of these materials to exhibit shear-thinning behavior—where viscosity decreases under shear—enables smooth extrusion during printing processes, while their high storage modulus and rapid recovery after shear ensure that the printed structure retains its shape and mechanical integrity once deposited. These are essential features for building stable constructs with high fidelity and load-bearing capacity, particularly in the context of regenerating cartilage or other mechanically active tissues. Moreover, the composition and concentration of the microparticles embedded within the ALMA matrix play a pivotal role in determining the viscoelastic behavior of the resulting granular hydrogels. A higher microparticle content generally enhances viscosity, stiffness, and recovery, but overly high concentrations can reduce structural reorganization capacity. The type of microparticles also made a difference, with CHIMA-based hydrogels, due to the rigid, positively charged chitosan backbone, tend to form stiffer, more robust structures suitable for applications requiring higher mechanical strength (e.g., cartilage or bone interfaces) and GelMA-based hydrogels, with a higher elasticity and recovery upon shear, making them potentially more suitable for softer tissue engineering applications where compliance and cell-friendly properties are prioritized.

### 2.5. CHIMA/ALMA and GelMA/ALMA Granular Hydrogel Biocompatibility and Chondrogenic Differentiation Potential

**Figure 6:**
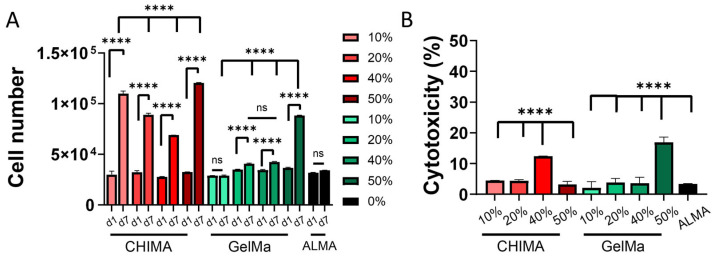
Cell proliferation and cytotoxicity in granular hydrogels. (**A**) Cell number as calculated from DNA assays within granular hydrogels at different microparticle concentrations after 1 or 7 days of culture. (**B**) Cytotoxicity normalized to cell number after 7 days of culture within granular hydrogels at different microparticle concentrations. Statistical significance was calculated from two-way ANOVA with post hoc Tukey’s multiple comparison tests; (****) *p* < 0.0001, ns = no significant difference.

To investigate the applicability of the developed granular hydrogels for cartilage tissue regeneration, first, the capability of the gels to support mouse mesenchymal stem cell (MoMSC) viability and proliferation over time was investigated. Cell proliferation and cytotoxicity of CHIMA/ALMA and GelMA/ALMA granular hydrogels with varying microparticle concentrations were evaluated after 1 and 7 days of culture using DNA quantification and lactate dehydrogenase (LDH) assays (Figure 6).

After 7 days of culture within granular and ALMA hydrogels, CHIMA/ALMA samples containing 10% and 50% microparticles demonstrated approximately three-fold increases in DNA content, indicating robust cell proliferation (Figure 6A). Interestingly, the 40% CHIMA group exhibited the lowest increase in cell number, suggesting that microparticle concentration has a non-linear effect on cell growth potential due to differences in microstructure or porosity affecting nutrient diffusion or cell–material interactions. In fact, a correlation between water absorption capability or swelling and weight loss (Figure 4D,E) with cell number appears evident. In all cases, there is a trend with increasing microparticle concentration from 10% to 40%, resulting in a lower swelling, lower weight loss, and lower cell proliferation. Moreover, this trend is disrupted at 50% CHIMA concentration, where swelling and cell proliferation values become higher than for 40% or 20% counterparts. This could be explained as a result of the packing density of the microparticles and the interactions with ALMA. As CHIMA microparticle concentration increases, the packing density becomes higher or more tortuous, and the porosity lower, hindering or at least making more difficult the diffusion of media and cells. At the highest microparticle concentration tested here (50%), the packing efficiency might decrease, leading to particle agglomeration, reducing tortuosity and, hence, facilitating the diffusion of media and cells. Despite this, the overall trend across all CHIMA-containing hydrogels was an increased DNA content from day 1 to day 7, indicating that CHIMA microparticles supported cell proliferation.

GelMA/ALMA hydrogels demonstrated the most substantial increase in DNA content (approximately twofold) in the 50% microparticle group. However, the extent of proliferation was generally lower in GelMA-based samples as compared to their CHIMA counterparts. In fact, CHIMA/ALMA hydrogels with 50% microparticles showed about 36% higher DNA content than GelMA/ALMA hydrogels at the same microparticle concentration by day 7. This could be attributed to differences in the mechanical and surface properties of CHIMA and GelMA microparticles, with CHIMA presenting a stiffer substrate for proliferation than GelMA.

These findings suggest that higher microparticle concentrations, particularly 50%, provide more surface area for cell attachment and spreading, thereby facilitating proliferation, as long as the overall structural environment remains permissive to cell infiltration and nutrient exchange. Moreover, the superior performance of CHIMA over GelMA in supporting proliferation may relate to its higher mechanical stiffness and potential for electrostatic interactions with cell membranes.

Cytotoxicity was calculated in all different hydrogel compositions after 7 days of culture and normalized to the cell number (Figure 6B). The results demonstrate that while the majority of samples exhibited low cytotoxicity, hydrogels of CHIMA and GelMA at 40% and 50% microparticle loading, respectively, showed a significantly higher cytotoxicity of approximately 15%. The materials used here crosslink by exposure to light, and no small-molecule-based crosslinkers are used. Thus, no side-products should be left on the system. Degradation products at this time-point and physiological conditions can only be attributed to uncrosslinked ALMA, GelMA, or CHIMA, and these are non-cytotoxic. Thus, we hypothesized that the slight increase in cytotoxicity may be attributed to the denser packing and more compact internal structure of these hydrogels at higher microparticle concentrations. Such dense packing can cause cell stress during encapsulation or seeding. Nevertheless, according to the ISO 10993-5 standard, a material is considered non-cytotoxic if cell viability exceeds 70%, which corresponds to a maximum acceptable cytotoxicity of 30% (533). All tested groups remained well below this threshold, and thus, all hydrogel formulations can be classified as biocompatible.

**Figure 7:**
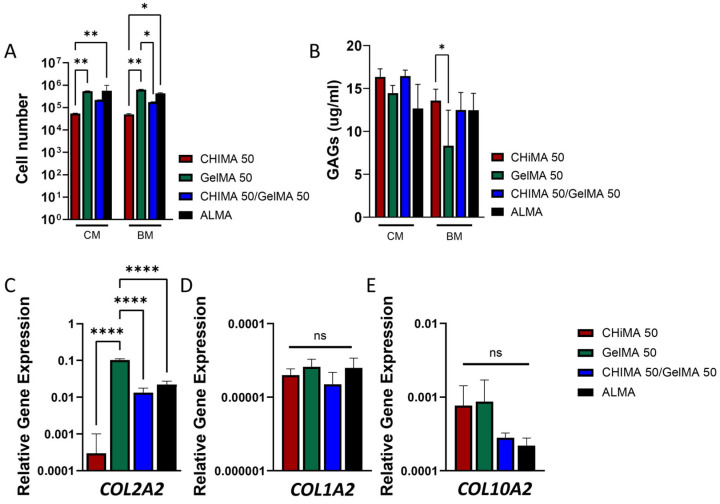
(**A**) Cell number as calculated from DNA assays in granular hydrogels cultured for 28 days in chondrogenic (CM) or basal (BM) media conditions. (**B**) Concentration of GAGs deposited by MoMSCs cultured for 28 days in granular hydrogels and ALMA hydrogels. Relative Col2a2 (**C**), Cola1a1 (**D**), and Col10A2 (**E**) expression in granular and ALMA hydrogels after 28 days of culture Statistical significance in A and B was calculated from one-way ANOVA with post hoc Tukey’s multiple comparison tests and in C-E from two-way ANOVA with post hoc Tukey’s multiple comparison tests; (****) *p* < 0.0001, (**) *p* < 0.01, and (*) *p* < 0.1. ns = no significant difference.

The ability of CHIMA/ALMA and GelMA/ALMA granular hydrogels to induce chondrogenic differentiation of MoMSCs was studied after 28 days of culture in chondrogenic and basal media conditions. To do so, we selected granular hydrogels composed of CHIMA/ALMA, GelMA/ALMA, and a composite hydrogel containing a 1:1 ratio of CHIMA and GelMA microparticles, under the assumption that the combination of both types of microparticles will provide cell adhesivity and high porosity (GelMA microparticles) while still increasing the overall rheological properties (CHIMA microparticles). Granular hydrogels with a 50% microparticle loading also presented the highest cell number after 7 days of culture, a swelling below the 20%, and the lowest weight loss or higher stability during culture periods of 72 h. Samples at 50% microparticles loading also displayed the highest storage module and shape recovery values over 90%. Thus, the total microparticle concentration was fixed at 50% for all CHIMA/ALMA, GelMA/ALMA, and 50:50 CHIMA:GelMA/ALMA hydrogels. Cell-laden hydrogels were cultured for 28 days under basal (BM) and chondrogenic (CM) media conditions (Figure 7). Initially, 15.000 cells were seeded per hydrogel and, after 28 days of culture, a general increase in DNA content was observed across all groups of over 1–2 orders of magnitude. Among all formulations, GelMA/ALMA hydrogels supported the highest levels of DNA content at day 28 in both basal and chondrogenic media, with a two-order-of-magnitude increase in DNA content. This enhanced proliferative response may be attributed to the microstructural features of GelMA-based granular hydrogels, which tend to exhibit a less densely crosslinked and more compliant network and are likely more permissive to cell migration and proliferation compared to the denser CHIMA-based matrices. In contrast, cells within CHIMA/ALMA hydrogels showed an increase of one order of magnitude, lower than that of GelMA hydrogels, which could be attributed to the bioactive nature of CHIMA, a chemically modified chitosan derivative that may better mimic native cartilage extracellular matrix and thus favor early chondrogenic differentiation at the expense of proliferation. Comparison with early measurements of cell number and proliferation at only 7 days of culture in basal media conditions (Figure 6) showed the opposite trend, with CHIMA/ALMA hydrogels presenting a higher cell number than GelMA/ALMA hydrogels. This effect can be ascribed to the stiffer nature of CHIMA microparticles that initially promote a faster proliferation, but at long term, the potentially less porous structure might hinder cell proliferation to the extent observed for GelMA hydrogels.

Glycosaminoglycans (GAGs) are essential components of the extracellular matrix (ECM) in cartilaginous tissues and are considered a marker of successful chondrogenic differentiation. These long, unbranched polysaccharides, such as chondroitin sulfate, keratan sulfate, and hyaluronic acid, play a crucial role in maintaining the structural integrity, hydration, and mechanical resilience of cartilage. They are secreted primarily by differentiated chondrocytes or mesenchymal stem cells undergoing chondrogenic differentiation and are typically embedded within a collagen type II-rich matrix. The sulfated nature of GAGs enables them to bind large amounts of water, which is vital for the compressive strength and viscoelastic behavior of cartilage. Additionally, GAGs interact with growth factors and cytokines in the microenvironment, helping regulate cell signaling, proliferation, and matrix assembly. The amount of GAGs produced in the granular hydrogels after 28 days of culture was overall higher in samples cultured in chondrogenic media, as expected (Figure 7B). Within media conditions, GAG deposition was highest in CHIMA/ALMA and CHIMA/GelMA/ALMA hydrogels compared to GelMA/ALMA or ALMA alone. This elevated GAG production is consistent with the lower DNA content observed in CHIMA and CHIMA/GelMA containing samples, suggesting that the cells have transitioned from a proliferative to a differentiative state.

To further evaluate the chondrogenic potential of the granular hydrogels, the expression levels of key cartilage and bone-related genes were analyzed after 28 days of culture using RT-PCR. Specifically, we assessed the expression of Col2a1 (type II collagen), Col10a1 (type X collagen), and Col1a1 (type I collagen) in all groups cultured in either basal or chondrogenic media (Figure 7C–E). Col2a1 is a widely recognized marker of hyaline cartilage and is indicative of early to mid-stage chondrogenic differentiation, as it is a major structural component of the cartilage extracellular matrix (ECM). Col10a1 is associated with hypertrophic chondrocytes and is often used to assess progression toward endochondral ossification or late-stage chondrogenesis. In contrast, Col1a1 is the primary collagen found in bone and fibrous tissue and serves here as a control to evaluate whether cells are diverting toward an undesired osteogenic or fibroblastic lineage.

The results revealed a substantial upregulation of Col2a1 expression in GelMA-containing hydrogels cultured in chondrogenic medium, suggesting a capacity to support chondrogenic differentiation. In contrast, Col2a1 expression was lower in CHIMA/ALMA hydrogels cultured under the same conditions. Hydrogels composed only of the supporting polymer ALMA also exhibited a relatively high level of Col2a1 expression, comparable to that of CHIMA/GelMA samples, suggesting that ALMA provides a permissive environment for chondrogenic differentiation of MoMSCs. The expression of Col1a1 was markedly lower across all hydrogel conditions when compared to Col2a1 and Col10a1 expression levels, indicating a limited activation of the osteogenic lineage and suggesting that the granular hydrogels support chondrogenic differentiation over osteogenesis. Nevertheless, while the expression level of Col1a1 was low for cells cultured in all hydrogel conditions, the expression levels of Col10a1 were lower (although not statistically significant) in samples of CHIMA/GelMA/ALMA and ALMA alone than in CHIMA/ALMA or GelMA/ALMA groups. Thus, the higher expression of Col10a1 in samples CHIMA/ALMA and GelMA/ALMA suggests that at longer culture periods, the initial onset of chondrogenesis observed as expression of Col2a1 and GAG deposition, could further evolve towards hypertrophic phenotypes with a higher expression and deposition of Col1a1 and Col10a1. This result suggests that the encapsulated MoMSCs predominantly remained in an early to mid-stage of chondrogenic differentiation, without progressing toward hypertrophy in the particular CHIMA/GelMA/ALMA hydrogels.

Maintaining a stable, non-hypertrophic chondrogenic phenotype is crucial for mimicking native articular cartilage and preventing ossification-related complications, which is a key goal. Thus, these findings are promising for cartilage tissue engineering, indicating the potential of CHIMA-containing microparticle hydrogels to support early chondrogenic differentiation for cartilage regeneration. Nevertheless, a deeper study of the cell phenotypic state and matrix deposition would be required to unravel the full potential of the developed granular hydrogels. Next steps would require assessing the impact of age, donor, and sex dependency on human cell differentiation and matrix deposition. Further in vivo studies would also be key to determining their applicability for the regeneration of cartilage.

## 3. Materials and Methods

### 3.1. Synthesis of GelMA, CHIMA, and ALMA

Methacrylated chitosan (CHIMA) was synthesized by reacting chitosan (CS Mw 600,000 to 800,000 Da, Thermo Scientific, Madrid, Spain, 1.035 g) with methacrylic anhydride (MA, Sigma Aldrich, Madrid, Spain, 6 mL) following a previously reported protocol [38]. In brief, a 1.5% (*w*/*v*) chitosan (100 mL) solution was prepared by dissolving a measured volume of a stock 4% (*v*/*v*) chitosan in acetic acid solution in PBS in a round-bottom flask at room temperature over 12 h. Methacrylic anhydride was gradually added to the chitosan solution, and the mixture was then maintained at 40 °C with gentle stirring at 60 rpm for 12 h, protected from light. The reaction product was precipitated by centrifugation, redispersed in deionized water, and purified by dialysis using a 10 kDa molecular weight cut-off membrane (SankeSkin, Thermo Fisher, Madrid, Spain) against 1 L of deionized water, with four water changes over a period of 72 h. The final product was then freeze-dried.

Methacrylated gelatin (GelMA) was synthesized using 1 g of gelatin (gelatin from porcine skin, gel strength 300, type A, Sigma Aldrich, Madrid, Spain) dissolved in 50 mL of distilled water for 3 h at 50 °C. A total of 0.25 mL of MA was diluted in 0.25 mL of dimethyl sulfoxide (DMSO, Fisher Bioreagents, Madrid, Spain), and 0.25 mL of the prepared mixture was added to the gelatin solution at 50 °C, under stirring at 800 rpm. The reaction was maintained for 3 h, adjusting the pH to 8–9 by adding 0.5 M NaOH (VWR Avantos, Barcelona, Spain) solution. After the reaction, the methacrylated product was diluted in deionized water, then purified via dialysis with a 10 kDa molecular weight cut-off membrane against 1 L of deionized water, with the water being refreshed four times over 72 h. Following purification, the final product was lyophilized.

To synthesize alginate methacrylate (ALMA), 1 g of alginate (alginic acid sodium salt from brown algae, medium viscosity, Sigma-Aldrich, Madrid, Spain) was dissolved in 100 mL of distilled water at 50 °C for 3 h. Next, 300 µL of methacrylic anhydride was added to the alginate solution, which was stirred at 800 rpm and maintained at 50 °C. The reaction was left to proceed for 3 h, adjusting the pH to between 8 and 9 by addition of 0.5 M NaOH.

### 3.2. Fourier-Transform Infrared Spectroscopy (FT-IR)

The functionalization of CHIMA was validated on an Alpha FT-IR (Bruker, Barcelona, Spain) spectrometer (Figure S2). Freeze-dried samples were scanned 32 times after air background correction from 400 to 4000 cm^−1^ and a step of 2 cm^−1^.

### 3.3. H-NMR Spectroscopy

ALMA and GelMA were characterized chemically by H-NMR (Figures S3 and S4). Samples were prepared by diluting 4–5 mg of polymer in D_2_O and analyzed on a Bruker NMR 500 MHz Advance spectrometer (Bruker BioSpin, Rheinstetten, Germany). In total, 128 scans were run per measurement. The efficiency of each reaction and the degree of methacrylation (DoM) of the resulting polymers were determined by peak integration. The DoM of GelMA was calculated using the ratio of the integral values of the aromatic ring in gelatine (7.5–7.6 ppm) that remain unchanged during reaction to the peaks of lysine (2.9–3.1 ppm), following Equation (1).

(1) $$DoM\;(\%)=(1-\frac{I\;GelMA\;lysine/methacrylic}{I\;gelatin\;lysine/methacrylic})\times 100$$

The *DoM* of GelMa oscillated between 30 and 35%.

The degree of methacrylation of ALMA was calculated as the ratio between the protons H5 and H1 of the glucuronic and manuronic units, respectively, appearing at positions 4.9–5.2 ppm and the signals of the vinyl protons of the methacrylate at 6.6 and 6.2 ppm [39]. Alternatively, the protons of the methyl group of the methacrylate were used to validate the results. Thus, ALMA yielded a DoM that varied between 2 and 6%.

### 3.4. Production of Chitosan Microparticles

Chitosan was dissolved in a solution of 1% (*v*/*v*) acetic acid at varying concentrations of 1%, 2%, 5%, and 8%. The methodology followed was adapted from a previously established approach by Rodriguez et al. [28]. In brief, chitosan solutions were introduced into a cottonseed oil bath containing Span 80 as a surfactant. Various experimental conditions were tested to optimize the formation of microparticles (Table 2 and Table S1). The goal was to produce well-defined microparticles that were completely separated without agglomeration and were of sufficient size to create adequate void spaces among the particles. To enhance the mechanical integrity of the chitosan microparticles, sodium citrate (Sigma Aldrich) (Table S1) or tripolyphosphate (TPP, Sigma Aldrich) (Table 2) was utilized as an ionic crosslinker. Microparticles crosslinked with TPP were emulsified overnight and crosslinked with 32% TPP for 7 h with an oil:surfactant ratio of 100 (*v*/*v*).

Once the microparticles were formed, they were separated from the oil phase using vacuum filtration. The microparticles were washed with a solution of acetone diluted with water in a 1:4 ratio. This washing step ensured that any residual oil or surfactant was removed from the surface of the microparticles. Finally, the chitosan microparticles were air-dried at room temperature.

### 3.5. Production of GelMA Microparticles

To prepare gelatin methacrylate (GelMA) microparticles as a control for comparison with other types of microparticles, a specific protocol was followed. Initially, GelMA was dissolved in distilled water at a concentration of 30% (*w*/*v*). From this solution, 3.3 mL were then carefully dropped into 16.5 mL of paraffin oil containing 165 µL of Span 80 surfactant to stabilize the emulsion, under continuous stirring at 800 rpm. The emulsification process was maintained for 15 min, allowing the GelMA solution to disperse into fine droplets within the oil phase. Following emulsification, the mixture was transferred to an ice bath (0–5 °C) for 30 min to facilitate solidification of the microparticles, with continuous stirring at 800 rpm. This cooling step was essential to ensure the microparticles retained their shape and integrity without further crosslinking. After solidification, 20 mL of acetone was added to the suspension to precipitate the GelMA microparticles, helping them further separate from the oil phase. Finally, vacuum filtration was employed to collect and isolate the microparticles from the oil, while an additional acetone wash was applied to remove any remaining oil residues. The resulting GelMA microparticles were then air-dried at room temperature.

### 3.6. Scanning Electron Microscopy (SEM)

The morphology and size distribution of the prepared microparticles were assessed using a TM3030Plus SEM (Hitachi High-Technologies, Tokyo, Japan) apparatus at different magnifications operating at 15 keV. The freeze-dried particles were deposited on a double-sided carbon sticky tape mounted on top of an aluminum holder and gold-coated using an SC7620 mini sputter coater/glow discharge system (Quorum, San Jose, CA, USA).

### 3.7. Fabrication of ALMA-Supported CS, CHIMA, and GelMA Granular Hydrogels

CS, CHIMA, and GelMA microparticles were utilized in the development of granular hydrogels, with ALMA (4% *w*/*v*) hydrogel serving as a structural support polymer. To create the hydrogels, dry microparticles from each group at varying concentrations (5%, 10%, 20%, 40%, and 50% *w*/*v*) were mixed with ALMA (4% *w*/*v*) solution containing 0.5% (*w*/*v*) lithium phenyl 2,4,6 trimethyl (LAP) photoinitiator. After mixing, the hydrogels underwent a dual-crosslinking process to further reinforce the interactions between microparticles. This process included an ionic crosslinking step in which samples were immersed for 5 min in calcium chloride (0.2 mM) and subsequently exposed to UV light for 10 min, both of which contributed to strengthening the crosslinking, ensuring the stability and robustness of the granular hydrogel network.

### 3.8. Swelling and Hydrogel Stability Studies

Hydrogel samples were prepared by freeze-drying and further weighed to obtain the initial dry weight, denoted as W_0_. To validate the stability of the hydrogels in terms of weight loss in water, the samples were immersed in water for 72 h. After this period, the hydrogels were freeze-dried again and weighed to determine the swollen dry weight, W_1_. The swelling ratio was then calculated using the formula:

$$\mathrm{Swelling}\;\mathrm{Ratio}\;=\;\frac{W_{1}-W_{0}}{W_{0}}\times 100$$

This measurement provided insight into the hydrogels’ capacity for water uptake and stability in hydrated conditions.

### 3.9. Rheological Characterization

The rheological characterization was performed using a Thermo Scientific Haake RheoStress1 rheometer (IFI, Vigo, Spain), configured with a 35 mm diameter serrated plate–plate setup and a 1 mm gap for all measurements. The temperature was maintained at 25 °C throughout the experiments. To determine the linear viscoelastic region (LVR), amplitude sweep tests were first conducted within a strain range (e.g., 0.001–10%) at a constant frequency (typically around 1 Hz), establishing the maximum strain that could be applied without altering the viscoelastic response. Once the LVR was identified, an optimal strain value of 0.01% was selected for subsequent oscillatory measurements. Frequency sweep tests were then conducted over a range of 0.1 to 100 Hz at a constant strain within the LVR. Rheological parameters, specifically the storage modulus (G′) and loss modulus (G″), were recorded as a function of frequency. Additionally, viscosity as a function of frequency was measured to assess the hydrogel’s viscoelastic properties. The rheological assessment was conducted on samples both before and after crosslinking treatments using UV light and CaCl_2_. To assess the stress relaxation properties of the granular hydrogels, viscosity measurements were conducted as a function of time. Using the same Thermo Scientific Haake RheoStress1 rheometer, a constant strain within the linear viscoelastic region (LVR) was applied to the hydrogel samples. Measurements were performed at 25 °C under constant strain and constant frequency, monitoring the viscosity over a period of 300 s to evaluate the gels’ time-dependent relaxation behavior.

### 3.10. Cell Culture and Encapsulation in CHIMA/ALMA and GelMA/ALMA Granular Hydrogels

Mouse mesenchymal stem cells (MoMSCs, ATCC-CRL-12424 D1 Orl Uva bone marrow stromal cell line) were expanded in monolayer culture at a density of 3.3 × 10^3^ cells per cm^2^ and in DMEM (Gibco™, Thermo Fisher Scientific, Basel, Switzerland) supplemented with 10% (*v*/*v*) fetal bovine serum (FBS, Corning Media Tech, Wiesbaden, Germany) at 37 °C with 5% of CO2 until 80% confluence. The culture media were changed every 2 days. Phosphate-buffered saline (PBS) was used to wash adherent cells before detachment with 0.25% Trypsin/EDTA solution (Gibco™).

Microparticles were sterilized by exposing the dry powder for 1 h to UV light [40], while ALMA solutions were sterilized by filtration through 0.2 µm pore diameter syringe filters. To create cell-laden granular hydrogels, MoMSCs dispersions in passage 9 (still retaining stemness and proliferative capacity [41]) were prepared at a concentration of 3 × 10^5^ cell/mL of sterile microparticle/ALMA dispersions, ensuring a high cell density for optimal cell-to-cell interaction and viability. Cell-laden ALMA hydrogels were used as a control. The cell–polymer suspensions were then pipetted into 12-well plates and crosslinked by 10 min exposure to UV light. Afterwards, cell culture media (α-MEM supplemented with 10% *v*/*v* FBS) was added to the wells.

### 3.11. DNA Assay

To assess total DNA content, the CyQuant™ cell proliferation assay (Invitrogen, Paisley, Scotland) was employed according to the manufacturer’s guidelines, with an assumption of 6.6 × 10^−6^ μg of DNA per cell to estimate cell numbers [42]. MoMSC-containing granular hydrogels were retrieved after 1 and 7 days of culture, followed by three washes with DPBS to remove residual media. Dry samples were then freeze–thawed three times to disrupt the cells and subsequently digested overnight at 56 °C using Proteinase K (1 mg/mL, Fisher BioReagents™, Illkirch-Graffenstaden, France) in Tris/EDTA buffer. For quantification, a standard curve was prepared with serial dilutions of the CyQuant™ DNA standard, ranging from 2 μg/mL down to 0 μg/mL. To remove RNA interference, an RNase-containing lysis buffer, diluted at 1:500, was added in equal volume to each sample, mixed, and incubated at room temperature for 1 h. In parallel, the GR-dye stock solution was diluted 200-fold into the lysis buffer. Following this, 100 μL of each sample was pipetted in triplicate into wells of a black-bottom microplate. Then, 100 μL of the GR-dye solution was added to each well, and after 15 min of incubation at room temperature, fluorescence was measured at 480 nm excitation and 520 nm emission.

### 3.12. LDH Cytotoxicity Assay

The LDH Cytotoxicity Assay offers a reliable and easy-to-use colorimetric method for assessing cell damage. Lactate dehydrogenase (LDH), a cytoplasmic enzyme present in numerous cell types, is released into the culture medium when cell membrane integrity is compromised. To test the biocompatibility of the granular hydrogels, cell-laden hydrogel samples were removed from culture following a 24 h incubation. The CyQUANT™ LDH Cytotoxicity Assay kit was employed according to the manufacturer’s protocol. In brief, to determine Maximum LDH Activity, 10 µL of 10×X Lysis Buffer was added to cells cultured on a plastic surface and incubated at 37 °C with 5% CO_2_ for 45 min.

For each sample, 50 µL of medium (representing Spontaneous LDH Activity, Maximum LDH Activity, and the LDH activity of cells embedded in granular hydrogels) was transferred into a 96-well flat-bottom plate. Next, 50 µL of the reaction mixture was added to each well and incubated for 30 min in the dark. Afterward, 50 µL of the stop solution was introduced to each well and gently mixed. Absorbance readings were taken at 490 nm and 680 nm, and cytotoxicity levels were calculated using the following formula:

$$\%\;\mathrm{Cytotoxicity}\;=\;\frac{Compound-treated\;LDH\;activity-Spontanous\;LDH\;activity}{Maximum\;LDH\;activity-Spontanous\;LDH\;activity}\times 100$$

### 3.13. Cell Differentiation Experiments

After evaluating the cell viability and biocompatibility of CHIMA/ALMA and GelMA/ALMA granular hydrogels, the microparticle concentration of 50% was chosen for carrying out chondrogenic differentiation assays. For this purpose, cell-loaded CHIMA/ALMA and GelMA/ALMA granular hydrogels were developed as described before. The prepared samples were incubated in basal medium (α-MEM supplemented with 10% *v*/*v* FBS) for 24 h and then this was substituted with the differentiation medium (high glucose (4.5 mg mL^−1^) DMEM (Dubelcco’s Modified Eagle Medium, Gibco) with 100 μg mL^−1^ of sodium pyruvate (Gibco), 0.2 mM L-ascorbic acid-2-phosphate (ASAP, Sigma-Aldrich), 1% 100× ITS liquid media supplement (Thermo Fisher Scientific), 40 μg mL^−1^ proline (Sigma-Aldrich), 100 U mL^−1^ penicillin/streptomycin (P/S), and 100 nM dexamethasone (Sigma-Aldrich). To the complete media and right before addition to the cultures, 0.01 ng mL^−1^ of TGF-β1 was supplemented (PeproTech, Rocky Hill, NJ, USA). Samples incubated in the basal medium were used as a control. The media was changed every 2–3 days for both basal and differentiation conditions. After 28 days, DNA assay, LDH, GAGs, and real-time PCR (RT-PCR) analyses were performed.

### 3.14. Glycosaminoglycan (GAG) Quantification

To evaluate the glycosaminoglycan content, the same digested samples obtained from the proteinase K treatment used in the DNA assay were analyzed. A colorimetric assay based on 1,9-dimethyl-methylene blue (DMMB) zinc chloride double salt (Sigma-Aldrich; prepared by dissolving 16 mg DMMB in 5 mL ethanol) was employed. For each measurement, 150 μL of the DMMB reagent was combined with 25 μL of the sample and 5 μL of 2.3 M NaCl in a black 96-well plate. The assay relies on the metachromatic shift in DMMB upon binding to sulfated GAGs, and the absorbance difference was recorded at 525 nm and 595 nm to enhance specificity. The GAG concentration in each sample was calculated using a standard calibration curve generated from known concentrations of chondroitin sulfate (Sigma-Aldrich).

### 3.15. Gene Expression Analysis: RNA Isolation and Purification, cDNA Synthesis and RT-PCR

After 28 days of culture in basal and chondrogenic media, the cell-laden hydrogels were rinsed with PBS three times. To lyse the cells, hydrogels were covered with 1 mL of TRIzol for 5 min at room temperature and underwent three cycles of freezing–thawing using liquid nitrogen. To remove proteoglycans, which can strongly inhibit the efficiency of PCR, an additional centrifugation step was performed at 12,000× *g* for 5 min, facilitating their precipitation. The resulting supernatant was then combined with 200 μL of chloroform (CHCl_3_), shaken vigorously for 15 s, and left to incubate at room temperature for 3 min. This was followed by centrifugation at 12,000× *g* for 15 min. The clear upper phase was carefully collected and mixed with 500 μL of isopropanol, then incubated for 10 min at room temperature. A subsequent centrifugation at 12,000× *g* for 10 min at 4 °C allowed RNA to precipitate into a gel-like form. This RNA pellet was then resuspended in an equal volume of ethanol for further purification. The RNA extraction process continued according to the Invitrogen™ PureLink™ RNA Mini Kit protocol, ensuring high-quality RNA elution. The RNA concentration was determined using an IMPLEN NanoPhotometer N60/N50, based on absorbance at 260 nm. Purity was assessed by examining the A260/A280 and A260/A230 absorbance ratios.

For complementary DNA (cDNA) synthesis, the Applied Biosystems™ (Life Technologies Europe, B.V., Bleiswijk, The Netherlands) High-Capacity RNA-to-cDNA™ Kit was used in a 20 μL reaction volume, applying the thermal profile of 37 °C for 60 min, followed by 95 °C for 5 min. In the qPCR experiment, 10 μL of 100 μM forward and reverse primers for Col1a1, Col2, and Col10 (Table 3) were diluted with 980 μL of molecular-grade water to yield a 1 μM solution. Each reaction contained 8 μL of the primer mix (0.4 μM), 2 μL of synthesized cDNA at 100 ng/µL yielding a final concentration of 10 ng/μL, and 10 μL of SYBR Green 2× Master Mix, all added to a 96-well clear reaction plate. Amplification was carried out using a QuantStudio 1 Real-Time PCR System (Applied Biosystems), using the following thermal cycling conditions: an initial step at 50 °C for 2 min, then 95 °C for 2 min, followed by 40 cycles of 95 °C for 15 s and 60 °C for 1 min. The cycle threshold (Ct) values for each sample were obtained and used in the comparative Ct (ΔΔCt) method to quantify relative gene expression levels, with GAPDH serving as the internal reference (housekeeping) gene.

### 3.16. Statistics

All data were plotted and statistically analyzed using the GraphPad Prism 9.0.2 software (La Jolla, CA, USA). In all cases, data normality was verified using the Shapiro–Wilk test, and a normal QQ plot was assessed before statistical analysis using parametric tests (*t*-test, ANOVA) and Tukey’s multiple comparisons tests. Data are presented as mean ± SD, and each individual point is provided in the plots. Additionally, the n number of each assay is provided in the figure legends. *p* values of <0.05 were considered statistically significant (ns *p* > 0.05; * *p* < 0.05; ** *p* < 0.01; and *** *p* < 0.001).

## 4. Conclusions

In this study, granular hydrogels composed of chitosan methacrylate (CHIMA), gelatin methacrylate (GelMA), and the support polymer (ALMA) were developed and characterized for their potential application in cartilage tissue engineering. The fabrication of CHIMA and GelMA microparticles was optimized using the oil emulsion method. Thereafter, granular hydrogels were fabricated using different microparticle loading and characterized in comparison to the ALMA-only hydrogels. The rheological analysis of granular hydrogels showed that CHIMA/ALMA hydrogels had significantly higher storage and loss moduli, indicating a more mechanically robust structure. Increasing the microparticle concentration improved mechanical strength and resistance to deformation. All hydrogels exhibited shear-thinning behavior, which is beneficial for tissue engineering applications. After crosslinking, CHIMA-based hydrogels displayed higher viscosity, suggesting stronger interactions within the network. CHIMA/ALMA and GelMA/ALMA granular hydrogels bearing a 50% microparticle concentration were selected for their higher stability during culture conditions and viscoelastic properties and were studied as candidates for cartilage tissue regeneration. To that end, CHIMA/GelMA/ALMA hydrogels bearing a 50:50 ratio of both microparticle types were also investigated. All materials proved biocompatible and enabled cell proliferation, with the highest cell numbers being measured in GelMA microparticle-containing hydrogels. CHIMA- and CHIMA/GelMA-based granular hydrogels induced the highest GAG deposition in MoMSCs after 28 days of culture. In contrast, samples containing GelMA microparticles displayed the highest Col2a1 expression, but the lowest Col10a1 expression was detected on CHIMA/GelMA/ALMA granular hydrogels. Thus, CHIMA/GelMA/ALMA granular hydrogels induced chondrogenic differentiation of MoMSCs, with high GAG deposition and Col2a1 expression and low Col10a1 expression, and appear as potential candidates for cartilage tissue regeneration. However, evaluation of their full potential would require further analysis of their biomechanical and regenerative properties in vivo.

## Figures and Tables

**Table 1:** Parameters tested to fabricate chitosan microparticles using tripolyphosphate (TPP) crosslinker.

Sample Code	Chitosan Concentration (%)	Oil-to-Sample Ratio	Oil-to-Surfactant Ratio	Stirring Speed (rpm)	Microparticle Size (Median, CI 95%)
TPP-1	2	5.6	100	1000	NM
TPP-2	5	5.6	100	1000	3.5 ± 0.1
TPP-3	5	1.0	100	600	8.7 (6.6, 11.0)
TPP-4	5	1.0	100	1000	7.1 (5.1, 9.7)
TPP-5	2	5.6	100	600	5.6 (4.9, 6.2)
TPP-6	2	5.6	100	300	5.2 (4.3, 5.8)
TPP-7	2	1.0	100	600	3.2 (2.6, 3.5)
TPP-8	8	1.0	100	600	3.9 (3.2, 4.9)
TPP-9	5	1.0	100	300	8.9 (7.7, 11.0)

**Table 2:** Viscoelastic properties of CHIMA/ALMA and GelMa/ALMA hydrogels. G′ and G″ are reported at 1 Hz.

Sample	Microparticle Concentration (%)	G′ (kPa)	G″ (kPa)	Recovery of Complex Viscosity After 60 s
ALMA	0	0.41	0.15	85.6
CHIMA/ALMA	10	33.0	7.5	84.1
CHIMA/ALMA	20	28.5	5.9	85.3
CHIMA/ALMA	40	40.3	8.1	91.6
CHIMA/ALMA	50	66.4	10.1	90.2
GelMA/ALMA	10	11.5	0.95	84.5
GelMA/ALMA	20	16.9	1.3	89.7
GelMA/ALMA	40	18.5	1.1	92.7
GelMA/ALMA	50	19.5	2.9	95.1

**Table 3:** Forward and reverse primers used for qPCR.

Gene	Fw (5′–3′)	Rv (5′–3′)
*GAPDH*	TGGCAAAGTGGAGATTGTTGCC	AAGATGGTGATGGGCTTCCCG
*COL1A1*	GCTCCTCTTAGGGGCCACT	CCACGTCTCACCATTGGGG
*COL2A1*	AGTACCTTGAGACAGCACGAC	GCTCTCAATCTGGTTGTTCAG
*COLXA1*	TTCTGCTGCTAATGTTCTTGACC	GGGATGAAGTATTGTGTCTTGGG

## Data Availability

The raw data supporting the conclusions of this article will be made available by the authors on request.

## Supplementary Materials

The following supporting information can be downloaded at: https://www.mdpi.com/article/10.3390/ijms27062889/s1.
