# Supplementary material for: Chitosan- and Gelatin-Based Composite Granular Hydrogels for Cartilage Tissue Regeneration
Source: Int J Mol Sci. 2026 Mar 23;27(6):2889. doi: 10.3390/ijms27062889 (PMC13026145; doi:10.3390/ijms27062889)
Supplement: Supplementary file 1 [file ijms-27-02889-s001.zip › ijms-4186387-supplementary.pdf]

Chitosan and gelatin-based composite granular hydrogels for cartilage tissue regeneration

Neda Kathami[1,2], *Pedro Guerrero*[3,4], *Koro de la Caba*[3,4], *Ander Abarrategi*[2,5,6] and *Sandra Camarero-Espinosa*[1,6*]

Afiliations:

[1]BioSmarTE Lab, POLYMAT, University of Basque Country UPV/EHU, Av. de Tolosa, 72, 20018, Donostia-San Sebastián, Spain.

[2]Center for Cooperative Research in Biomaterials (CIC biomaGUNE), Basque Research and Technology Alliance (BRTA), 20014 Donostia-San Sebastian, Spain

[3] BIOMAT Research Group, University of the Basque Country (UPV/EHU), Escuela de Ingeniería de Gipuzkoa, Plaza de Europa 1, 20018 Donostia-San Sebastián, Spain

[4] BCMaterials, Basque Center for Materials, Applications and Nanostructures, UPV/EHU Science Park, 48940 Leioa, Spain

[5] Department of Cell Biology and Histology, Faculty of Medicine and Nursing, University of Basque Country (UPV/EHU), 48940 Leioa, Spain

[6]IKERBASQUE, Basque Foundation for Science, 48009 Bilbao, Spain

*Author to whom correspondence should be addressed.

Table S1. Parameters tested to fabricate chitosan microparticles using sodium citrate (NC) crosslinker. *: Post-fabrication washing with acetone:water 1:7.

Sample number	Chitosan concentration (%)	Oil to sample ratio	Oil to surfactant ratio	Stirring speed (rpm)	Emulsification time (min)	Crosslinker	Crosslinker concentration (%)	Crosslinking time (h)
**NC1**	1	5.6	100	600	30	Na-citrate	32	2
**NC2**	2	5.6	100	600	30	Na-citrate	32	2
**NC3**	2	5.6	100	600	30	Na-citrate	40	2
**NC4**	5	5.6	100	600	30	Na-citrate	40	2
**NC5**	8	5.6	100	600	30	Na-citrate	40	2
**NC6**	8	5.6	200	600	30	Na-citrate	40	2
**NC7**	8	4.5	100	600	30	Na-citrate	40	2
**NC8**	8	4	100	600	30	Na-citrate	40	2
**NC9***	5	5.6	100	600	30	Na-citrate	40	2
**NC10**	8	1	200	100	15	Na-citrate	40	Overnight

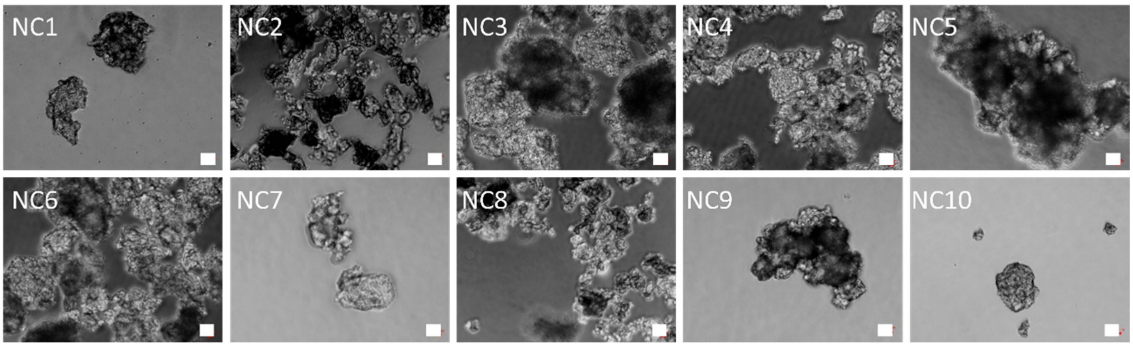

Figure S1. Chitosan microparticles prepared by emulsification under the process conditions defined in Table S1 using sodium citrate as crosslinker. Optical microscopy images of chitosan microparticles obtained from emulsification processes by varying the emulsion agitation speed, the oil-to-sample ratio, oil-to-surfactant ratio, crosslinker concentration and the initial polymer concentration. The scale bar in all images is 10 μm.

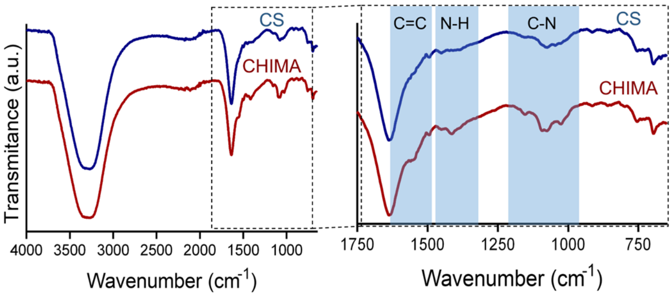

Figure S2. FT-IR spectra of chitosan (CS) and methacrylated chitosan (CHIMA). (Left) Full FT-IR spectra from 650 to 4000 cm-1. The dotted square box indicates the magnified area (right). Blue boxes indicate relevant areas of C=C, N-H, and C-N stretching. CHIMA spectra displayed a more intense peak at 1550 cm-1 that was ascribed to alkenyl C=C bond from the methacrylate, a peack at 1400 ascribed to N-H

stretching and a peak at 1070 cm-1 ascribed to C-N stretching from the linkage of the methacrylate to CS.[1]

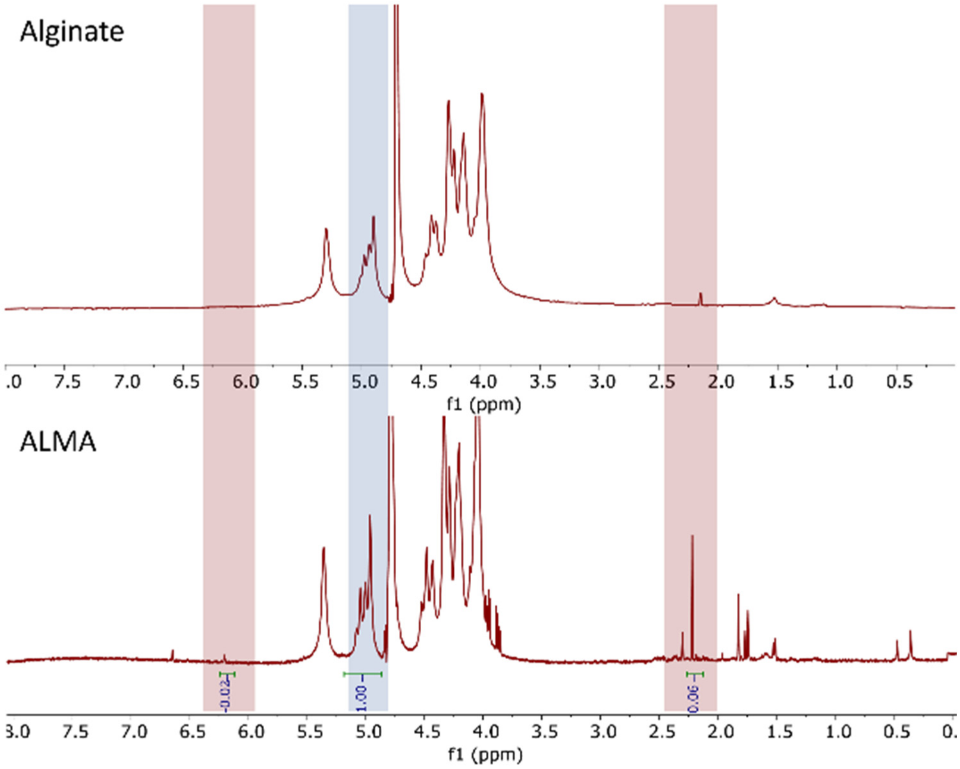

Figure S3. H1-NMR spectra of alginate and ALMA. The blue band indicates the region where H5 and H1 from glucuronic and manuronic units, respectively. The left red band coresponds to the vinyl proton of methacrylate groups used for quantification and the peak at 2.2 ppm cooresponds to the methyl groups of the methacrylate.[2]

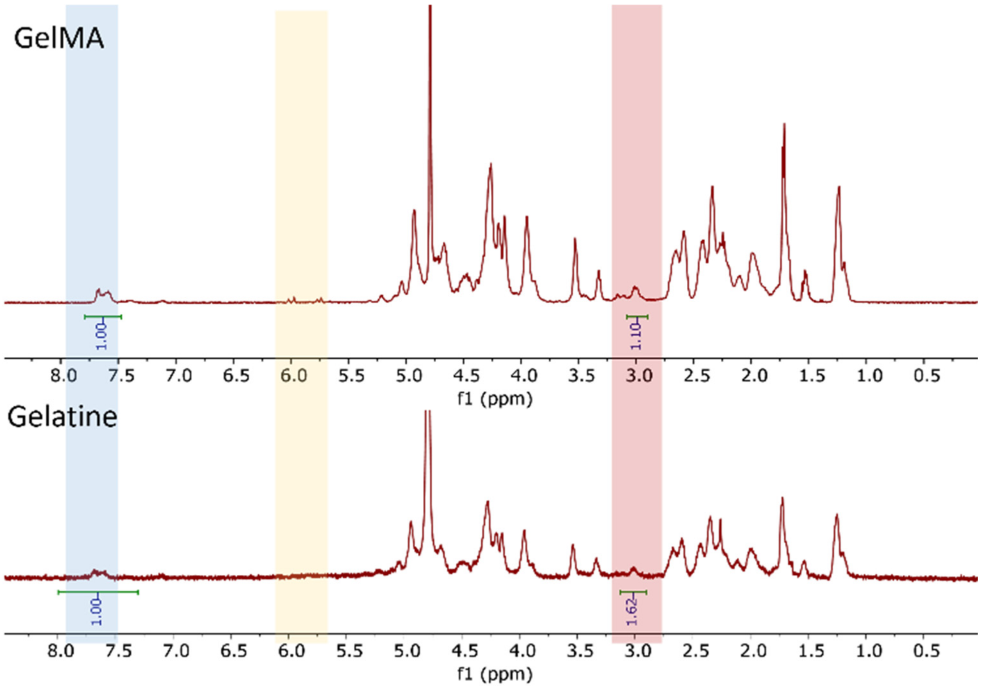

Figure S4. H1-NMR spectra of gelatine and GelMA. The blue band indicates the protons of the aromatic rings, the yellow band the protons of the vinyl from the methacrylate and the red, those belonging to lysine.

1. Morelli, S.; D'Amora, U.; Piscioneri, A.; Oliviero, M.; Scialla, S.; Coppola, A.; De Pascale, D.; Crocetta, F.; De Santo, M. P.; Davoli, M.; Coppola, D.; De Bartolo, L., Methacrylated chitosan/jellyfish collagen membranes as cell instructive platforms for liver tissue engineering. *International Journal of Biological Macromolecules* **2024,** 281, 136313.
2. Poshina, D.; Sokolova, N.; Nono-Tagne, S.; Ahmadi-Nohadani, H.; Gofman, I.; Mishanin, A.; Golovkin, A.; Skorik, Y.; Otsuka, I., Electrospinning of methacrylated alginate for tissue engineering applications. *RSC Advances* **2024,** 14, (52), 38746-38756.
